# Agility and Safety Performance among Nurses: The Mediating Role of Mindful Organizing

**DOI:** 10.3390/nursrep11030063

**Published:** 2021-08-30

**Authors:** Muhammad Shoaib Saleem, Ahmad Shahrul Nizam Isha, Yuzana Mohd Yusop, Maheen Iqbal Awan, Gehad Mohammed Ahmed Naji

**Affiliations:** 1 Department of Management & Humanities, Universiti Teknologi PETRONAS, Seri Iskandar 32610, Malaysia; shahrul.nizam@utp.edu.my (A.S.N.I.); maheen.iqbal90@gmail.com (M.I.A.); gehadnaji.utp@gmail.com (G.M.A.N.); 2 Faculty of Medicine, Universiti Sultan Zainal Abdin, Kampung Gong Badak 21300, Malaysia; yuzanayusop@unisza.edu.my

**Keywords:** mindful organizing, workforce agility, proactivity, adaptability, resilience, safety performance, nursing staff

## Abstract

This study aimed to assess the impact of workforce agility on private hospital nursing staff’s safety behavior with the mediating role of mindful organizing. This study was cross-sectional. A self-administered questionnaire was used to collect data from 369 nursing staff. The structural equation modeling (SEM) technique was used to check the internal consistency, convergent validity, discriminant validity, and hypotheses testing. For mediation analysis, the bootstrapping technique was used. Our findings suggested that workforce agility is the possible predictor of mindful organizing, as all of these dimensions have a positive impact on mindful organizing. Reference to safety performance sub-dimensions, proactivity, adaptability, and resilience had a positive significant impact on (a) safety compliance, and proactivity had a positive impact on (b) safety participation. Further, mindful organizing was also found to be positively associated with safety performance. Evidence for mediation between workforce agility and safety performance was also observed. Proactivity, adaptability, and resilience can enhance safety performance for the nursing staff. Workforce agility can also help the organization to attain mindful organizing, which will help them to achieve operational excellence, whereas in the past, high-reliability organizations were mainly found practicing mindful organizing. This study demonstrated the key impact of workforce agility and mindful organizing on safety behaviors directly and indirectly.

## 1. Introduction

The importance of workplace health and safety is paramount for all organizations, especially in the healthcare sector. The occupational health and safety phenomenon is still relevant globally, as the workforce in the healthcare sector is more prone to be exposed to different occupational hazards, i.e., exposure to chemicals, blood-borne pathogens, radiological activities, psychosocial factors, biological threats, and other ergonomic oddities while performing tasks. According to the United States Department of Labor, healthcare is the industry that has the highest injury rate as of 2017 with a total of 548,100 reported cases [1,2]. More importantly, nursing, one of the crucial elements of healthcare, is the only occupation that has the highest incidence rate, i.e., 10.9 (per 100 full-time workers) [1].

In the context of Malaysia, recent statistics from the Social Security Organization (SOCSO) exhibited that the healthcare sector registered 1549 cases overall in the year 2018 only. Further, the Social Security Organization (SOCSO) has paid compensation against 1365 cases of temporary disability, 463 cases of permanent disability, and 20 cases of death and mortality. In addition to this, 1344 individuals were the victim of chemical, physical, and biological hazards, and 1350 individuals were targeted by respiratory, skin, and musculoskeletal diseases/disorders [3] in different industrial sectors. Such statistics exhibit the worrying safety performance in the context of healthcare as well. Therefore, our research is particularly aimed to assess how occupational safety in the healthcare sector can be enhanced for its workforce.

Usually, safety performance at the workplace refers to such behaviors that workers undertake to comply and adhere to the predefined safety standards [4,5]. Consistent with the prior literature and keeping in view the difference between task and contextual performance, many authors have identified two dimensions of safety performance: safety compliance and safety participation [4,6]. Safety compliance is homogenous to task performance in terms of adhering to predetermined safety policies, protocols, and procedures [4]. Additionally, safety compliance refers to those behaviors that are mandated through organizational policies, i.e., adhering to SOPs, wearing PPEs, attending training, and following certain procedures in certain scenarios. In other words, safety behavior that requires formal enforcement and recognition falls under the domain of safety compliance [4]. Secondly, safety participation is more of an extra-role behavior, in which an employee supports overall safety processes, systems, and policies through voluntary participation [4]. Safety participation is most often not linked with formal recognition or rewards, where it is an intrinsic motivation and self-initiated actions of an employee that result in the contribution of overall safety performance in an organization [6]. A few examples of safety participation can be speaking up in meetings for safety, raising concern for safety while performing tasks, encouraging others to learn about safety, and supporting the overall safety policy of an organization [6]. Referring to the healthcare sector, contemporarily, much of the emphasis has been given to patient safety in terms of prescribing, diagnosis, and treatment [7], rather than factors that may influence employees’ safety performance. Our research will mitigate this literature bias by adding knowledge to healthcare workers’ safety behavior.

In the literature, studies have testified high-reliability organizations’ (HROs) principles, i.e., mindful organizing in healthcare settings previously, and were associated with decreased medication errors, patient falls [8,9,10], and fewer accidents [11]. On the other hand, much of the qualitative evidence is available, which creates a scarcity of quantitative evidence for mindful organizing [12], its antecedents, and its outcomes [13]. Mindful organizing is the collective ability of the workforce to detect anomalies and act swiftly [14]. Mindful organizing helps individuals to uncover small anomalies that can lead to catastrophic outcomes [15]. Five inter-related processes of mindful organizing (preoccupation with failures, commitment to resilience, reluctance to simplify instructions, sensitivity to operations, and deference to expertise) help individuals to make collective sense and foresee possible abnormalities and their anticipated outcomes [16]. The prior literature advocated mindful organizing as the main reason for error-free operations of high-reliability organizations (HROs) over an extensive period, even when accidents are inevitable in those systems [11,14]. According to the literature, antecedents of mindful organizing that foster, promote, and encourage this phenomenon are still unknown/underdeveloped [15,17].

From a theoretical and qualitative perspective, one of the possible antecedents of collective mindfulness is the proactivity of an individual [18]. Proactive individuals tend to have such strategies that enhance shared awareness about the situations to operate safely. Another possible antecedent of mindful organizing is the adaptability of organizations, where researchers have posited mindful organizing as an adaptive form of organization to act in an uncertain and ever-changing environment [19]. Tackling uncertainty demands organizations to be adaptable, which is only possible through an adaptive workforce, thus it would be interesting to see if individual adaptability can be the possible predictor of mindful organizing through empirical testing. Lastly, individual resilience can be the possible antecedent of mindful organizing. Resilient individuals tend to have a positive attitude against changes and new ideas, tolerance in dealing with situations, and they can cope with stress artfully [20]. Therefore, the resilience of an individual would also be tested as a possible antecedent of mindful organizing.

All in all, this research aims to test workforce agility’s sub-dimensions as a possible predictor of mindful organizing, as well as mindful organizing as a mediator between workforce agility (proactivity, adaptability, resilience) and safety performance (safety compliance and safety participation). Quantitative evidence relating workforce agility to safety performance is almost non-existent to date, which will bridge this knowledge gap.

## 2. Literature Review

### 2.1. Workforce Agility

For our study, workforce agility is interpreted as such behaviors of an employee that are agile, which is different from related concepts such as predispositions, agile personality, or different attributes. The classification of workforce agility and its sub-dimensions linked with attributes and behavior is based on a previous model posited by researchers such as Griffin, Hesketh, Dyer, and Shafer [21,22]. There are three main sub-dimensions of workforce agility: proactivity, adaptability, and resilience. As per the definition of proactivity, the proactivity of an individual refers to an initiation of actions that results in positive outcomes under a changing environment [21]. Examples of proactive behavior of employees are: forecasting the forthcoming problems based on change, proposing or even undertaking an action that can mitigate problems associated with the anticipated change, and preemptive actions that can resolve the associated problems.

The second dimension is adaptability, which is linked with one’s self, i.e., the modification of one’s own behaviors to suit the changing environment [21]. It comes into action when one is dealing with a different set of people having different backgrounds, experiences, environments, and equipment. Adaptability is also described as an individual’s ability to learn new knowledge, skills, and technologies. Adaptable individuals tend to be more flexible in terms of assuming new roles, working in different teams, and undertaking multiple tasks at the same time. The last dimension of workforce agility is resilience, which is how much the individual can cope with stressful events and their ability to recoup from the worst [23]. Resilient individuals tend to have a positive attitude toward their ongoings or even when they encounter failures. Based on the nature of the aforesaid dimensions of workforce agility, it would be interested to link them with mindful organizing. Therefore, individuals who have more proactive, adaptive, and resilient behavior are more likely to indulge in mindful organizing. Based on the literature and aforementioned arguments, the following hypotheses are formed to be tested:

**Hypothesis** **1** **(H1).** *Proactivity is positively associated with mindful organizing.*

**Hypothesis** **2** **(H2).** *Adaptability is positively associated with mindful organizing.*

**Hypothesis** **3** **(H3).** *Resilience is positively associated with mindful organizing.*

**Hypothesis** **4** **(H4).** *Proactivity is positively associated with safety compliance.*

**Hypothesis** **5** **(H5).** *Adaptability is positively associated with safety compliance.*

**Hypothesis** **6** **(H6).** *Resilience is positively associated with safety compliance.*

**Hypothesis** **7** **(H7).** *Proactivity is positively associated with safety participation.*

**Hypothesis** **8** **(H8).** *Adaptability is positively associated with safety participation.*

**Hypothesis** **9** **(H9).** *Resilience is positively associated with safety participation.*

### 2.2. Mindful Organizing

Mindful organizing is a relatively new and emerging concept. It was conceptualized in high-reliability organizations (HROs), e.g., nuclear power plants, nuclear submarines, aircraft carriers, air traffic management, and the aviation industry, i.e., how the salient processes of high-reliability organizations (HROs) facilitate continuous learning and adaption to the situation to safeguard from any catastrophe [14]. It is a social construct that prevails through teams and through which collectives can detect, discern, and act according to the situation [14]. Mindful organizing involves five principal processes:

- *Preoccupation with Failure* (detection of cues and thinking or possibility to go wrong). To overcome failures, one must embrace them and report them. In HROs, individuals report the smallest anomalies and are encouraged to see the larger picture of small events. Employees always consider the probability of things going wrong in their minds [14,24,25,26].
- *Reluctance to Simplify* (ability to ask, assess, and question the given assumptions and orders) is another key principle of high-reliability organizations. There is reluctance for simply absorbing or passing the issue at hand. Reluctant individuals are more skeptical about their surroundings and they do not perceive or absorb things easily [14,24,25,26].
- *Sensitivity to Operations* (situational awareness of the front-end employees and detection of an anomaly at an early stage). HROs are sensitive to their ongoing operations. This means that much importance is given to the front line where the actual work is taking place and it is directly related to the possible mishap. It is at the discretion of an individual to balance between sensitivity and predefined rules and regulations to adjust according to the situation being faced [14,24,25,26].
- *Commitment to Resilience* (capability to fight back from the adverse situation and normalize the overall environment). HROs can cope with adversities and return to normal operations quickly. Resilience is achieved through training mocks and drills in high-reliability organizations by reflecting worst-case scenarios and fabricated conditions to learn and adapt [14,24,25,26].
- *Deference to Expertise* (delegation of authority to those who are experts for the problem at hand). Decisions based on strict hierarchies are more prone to errors and can cause delay. Deferring authority does not essentially mean delegating authority to the most experienced individual who may be experienced but not expert, but delegating authority to the most relevant individual who possesses the expertise of that particular event. Knowledge, skills, and abilities are commonly shared and are known to the other team members [14,24,25,26].

Based on these arguments and in congruence with the prior literature [9,15,16], it would be valuable to relate mindful organizing to the objective indicators of safety performance (safety compliance and safety participation) amongst nursing staff, thus the following hypotheses are formed to be tested:

**Hypothesis** **10** **(H10).** *Mindful organizing is positively associated with safety compliance.*

**Hypothesis** **11** **(H11).** *Mindful organizing is positively associated with safety performance.*

**Figure 1:**
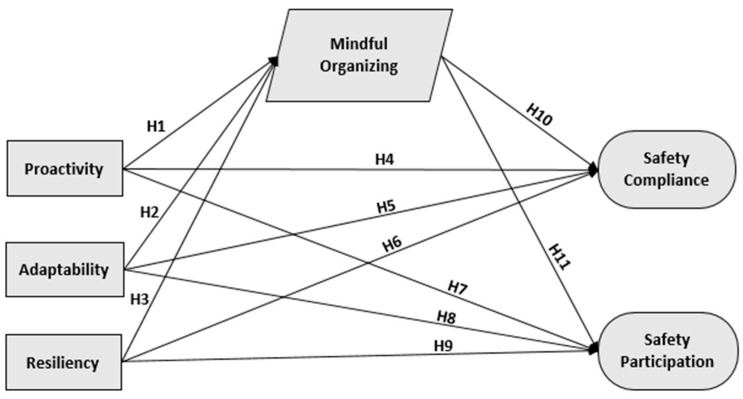
Proposed research framework.

Based on the theoretical explanation of the constructs being studied, Figure 1 is made to show the overall research framework.

## 3. Methods

### 3.1. Sample and Data Collection

For this study, we followed the guideline set forth by the American Psychological Association (APA), commonly used globally. We gathered the data from different private hospital nursing staff. All of those hospitals were located in different localities of the Malaysian Peninsula, i.e., eastern, western, northern, and southern regions. We used a convenience and snowball sampling technique. All nursing staff were provided with a copy of the questionnaire and participation in the survey was voluntary. Each respondent was part of a team and a permanent employee. Participants were encouraged to complete the questionnaire on the spot and necessary assistance was provided to them whenever it was needed. All ethical values and standards were catered for throughout the data collection, i.e., asking consent for participation and appraising the participation. The data were collected over five months from January to May 2021. Overall, 425 questionnaires were distributed, and after gathering all the filled survey forms, we performed initial screening (excluding missing values and unclear responses), thus finally including 369 survey responses in the final analysis—this makes an 87% (*n* = 369/425) response rate. The average team of nursing comprised 5 permanent members and a team is defined in terms of two or more employees working for the attainment of a mutual goal [27].

### 3.2. Measures Used

#### 3.2.1. Workforce Agility Scale

Workforce agility was measured through its English version developed by Sherehiy [28] and used by other researchers in the prior literature [20]. This scale is multidimensional, as it has three further sub-dimensions of proactivity, adaptability, and resilience. Firstly, the proactivity dimension comprised 11 items rated on (strongly disagree, 1) to (strongly agree, 5). The example statement of the proactivity dimension is, “I look for the opportunities to make improvements at work”. Secondly, the adaptability dimension comprised 13 items ranging from (strongly disagree, 1) to (strongly agree, 5). The example statement for the adaptability scale is, “Change my behavior to work more effectively with other people”. Lastly, the resilience dimension comprised 12 items ranging from (strongly disagree, 1) to (strongly agree, 5), and an example statement is “I am tolerant to situations where things seem confusing.”

#### 3.2.2. Mindful Organizing Scale

For this study, the scale developed by Weick and Sutcliffe [26] was used to measure mindful organizing among teams. The nature of this scale is unidimensional and it comprises nine items overall. With the help of this scale, it is possible to observe the extent to which work teams are being attentive to the forthcoming issues and how they act and plan accordingly [14,24]. Respondents were asked to rate their own and their team’s effort towards mindful organizing on a 5-point Likert scale ranging from (strongly disagree, 1) to (strongly agree, 5). An example statement of the questionnaire is, “We spend time identifying activities we do not want to go wrong”.

#### 3.2.3. Safety Performance Scale

Self-reported safety performance scales developed by Neal and Griffin [6] were used to assess employees’ safety behavior. These scales measure the extent to which employees adhere/comply with the safety standards as well as how much they participate in improving the overall safety performance of an organization. Overall, both dimensions of safety performance contain six items rated on a Likert scale ranging from (strongly disagree, 1) to (strongly agree, 5). Example statements for the safety compliance scale are, “I use all the necessary safety equipment to do my job,” and “I use the correct safety procedures for carrying out my job“. Some of the example statements for safety participation include, “I promote the safety program within the organization”, “I put in extra effort to improve the safety of the workplace” and “I voluntarily carry out tasks or activities that help to improve workplace safety”.

#### 3.2.4. Analysis

Before analyzing the study hypotheses, we conducted confirmatory factor analysis (CFA) to assess the distinctiveness of our study variables. Workforce agility and safety performance were measured at the individual level. Mindful organizing was assessed at the team level, and for that reason, we also tested within-team agreement by using interrater agreement indices to assess within-group agreement through r_wg(j)_ [29]. This index provides necessary information for justifying the aggregation of scores at a team level. For reliability, we used the Cronbach’s alpha coefficient, and for descriptive statistics, we used SPSS V.21. Overall structural equation modeling, hypotheses testing, and mediation analysis were conducted using AMOS-21. Both convergent and discriminant validities were assessed for each variable. Measures such as average variance extracted (AVE), standardized factor loading (SFL), and composite factor loadings (CR) were utilized to assess convergent validity. The goodness-of-fit for the overall model was assessed through indices such as the root-mean-square error of approximation (RMSEA), goodness-of-fit index (GFI), adjusted goodness-of-fit index (AGFI), normed fit index (NFI), comparative fit index (CFI), Tucker–Lewis index (TLI), CMIN (chi-square χ^2^/degree of freedom), and chi-square χ^2^ [30,31].

## 4. Results

### 4.1. Reliability, Validity, Aggregation, and Demographic Analyses

The internal consistency of each construct was tested to assess the overall reliability. As stated in the literature, a Cronbach’s alpha value greater than 0.70 indicates that the reliability is achieved [32,33]. For all of the research constructs, the Cronbach’s α coefficients were: proactivity (0.96), adaptability (0.95), resilience (0.94), mindful organizing (0.92), safety compliance (0.83), and safety participation (0.87). Such values exhibit sound reliability. Demographically, 310 respondents were female and 59 were male, with an overall average age of 35 years. The average working experience was eleven years with a minimum of 5 and a maximum of 22 years. From an educational perspective, 41% (*n* = 150/369) of respondents were diploma holders, 47% (*n* = 173/369) of respondents held a bachelor’s degree, and 12% (*n* = 46/369) of respondents held a master’s degree. Data were collected from two private hospitals in Perak, three in Johor, three in Kelantan, two in Negri Sembilan, two in Penang, and three in Selangor. Lastly, for aggregation analysis of mindful organizing, r_wg(J)_ values were above the cut-off limit, i.e., 0.70 [34], (r_wg(J)_ = 0.90), which shows strong agreement among team members.

Convergent validity results were assessed through construct reliability (CR), average variance extracted (AVE), and standardized factor loadings (SFL). The acceptance criteria for such matrices are CR > 0.7, AVE > 0.5, and SFL > 0.6. According to the results, all constructs were well under the predefined criteria and are depicted in Table 1 [35]. For discriminant validity, the correlation coefficient of all variables was compared with the square root of average variance extracted (AVE) [35], and the discriminant value was achieved accordingly (presented in Table 2).

### 4.2. Measurement Model

For the assessment of the quality of our measurement models for workforce agility, mindful organizing, and safety performance, absolute fit indices (RMSEA = root-mean-square error of approximation, GFI = goodness-of-fit), incremental indices (AGFI = adjusted goodness-of-fit index; NFI = normed fit index, TLI = Tucker–Lewis index, CFI = comparative fit index), and parsimonious normed-fit indices (NC = normed χ^2^, i.e., χ^2^/degree of freedom) were used [30,31]. Our extracted values were in harmony with the prescribed limits/criteria. Findings for the measurement models are displayed in Table 3, where it is obvious to see that our findings have reported an acceptable good fit against the confirmatory factor analysis (CFA).

### 4.3. Structural Model

Our hypothesized model (Figure 1) was assessed through the structural equation modeling (SEM) technique, whereas the overall data fit and structural model were estimated through the goodness-of-fit. Primarily, data were assessed to see if any abnormal variables existed [35,36,37]. According to the data, standard errors for all variables were significant and greater than zero with values less than 0.068, and all our standardized factor loadings (SFL) were also significant with values ranging from 0.72 to 0.86. All of the aforesaid findings report a good preliminary fit of our data. Finally, absolute, incremental, and parsimonious indices were used to check the overall model fit [30]. Further, all of the finding met the decided criteria (RMSEA = 0.015, GFI = 0.883, AGFI = 0.871, NFI = 0.910, TLI = 0.992, CFI = 0.993, χ^2^ = 1304.785, χ^2^/DOF = 1.079, *p* < 0.05), demonstrating an acceptable overall model fit.

### 4.4. Hypotheses Testing

Our hypothesized model (Figure 1) was assessed through the structural equation modeling (SEM) technique, whereas the overall model depicts a sufficient amount of prediction for safety performance as our predictors accounted for about 31% of variance in safety compliance and 49% of variance in safety participation. In addition to this, 14% of variance was explained by mindful organizing predictors. The proposed association of overall constructs was evaluated and is exhibited in Table 4. Initially, Hypotheses 1, 2, and 3 under the construct of workforce agility were tested. As estimated, proactivity H1 (β = 0.24, *p* < 0.001), adaptability H2 (β = 0.092, *p* < 0.05), and resilience H3 (β = 0.156, *p* < 0.001) had a positive and significant impact on mindful organizing, thus Hypotheses 1–3 were accepted. Secondly, Hypotheses 4–9 were tested for their direct impact on safety compliance and safety participation. As assumed early, H4 (β = 0.030 *p* < 0.001), H5 (β = 0.239 *p* < 0.001), H6 (β = 0.104 *p* < 0.001), and H7 (β = 0.0549 *p* < 0.001) had a positive and significant impact on both safety compliance and safety participation. However, H8 (β = 0.075 *p* > 0.05) and H9 (β = 0.044 *p* > 0.05) were rejected based on their nonsignificant *p*-value, thus Hypotheses 8 and 9 were not supported. Lastly, the direct effect of mindful organizing on both safety compliance and safety participation was tested. As expected, mindful organizing H10 (β = 0.249 *p* > 0.001) and H11 (β = 0.0158 *p* > 0.001) had a positive impact on both safety performance indicators, and consequently Hypotheses 10 and 11 were both supported. The overall path coefficient of the model is shown in Table 4.

### 4.5. Mediation Effect Assessment

Referring to the mediating effect of mindful organizing between workforce agility sub-dimensions and safety performance (safety compliance and safety participation), the results showed that proactivity (β = 0.372, *p* < 0.001), adaptability (β = 0.237, *p* < 0.001), and resilience (β = 0.112, *p* < 0.001) had a positive and significant direct impact on safety compliance. Notwithstanding, proactivity (β = 0.648, *p* < 0.001) only had a direct and positive impact on safety participation, whereas adaptability (β = 0.177, *p* > 0.05) and resilience (β = 0.045, *p* > 0.05) had a positive but non-significant impact on safety participation.

Pertaining to the assessment of the indirect effect of workforce agility’s sub-dimensions on safety compliance, a positive and significant impact was observed for proactivity (β = 0.073, *p* < 0.001), adaptability (β = 0.023, *p* < 0.05), and resilience (β = 0.041, *p* < 0.05). Additionally, regarding the assessment of the indirect effect of workforce agility’s sub-dimensions on safety performance, proactivity (β = 0.045, *p* < 0.05), adaptability (β = 0.041, *p* < 0.05), and resilience (β = 0.025, *p* < 0.05) had a positive and significant impact as well. From the aforementioned statistical findings, it was observed that mindful organizing partially mediates between proactivity, adaptability, resilience, (1) safety compliance, and (2) safety participation. Additionally, mindful organizing fully mediates between adaptability, resilience, and safety participation. It was also worthwhile to notice that the indirect effect of all predictors for safety performance was significant, showing that mediation is taking place. Table 5 depicts the overall mediation results.

## 5. Discussion and Conclusions

Through the study findings, overall support was attained for the proposed model. Workforce agility (proactivity, adaptability, and resilience) was anticipated as a possible predictor of mindful organizing. Further, mindful organizing is also related to safety participation and safety compliance. To be precise, we proposed the mediation of mindful organizing between workforce agility’s sub-dimensions and objective indicators of safety performance. The study findings supported our hypotheses except for two only (Adaptability → Safety Participation and Resilience → Safety Participation). Our findings also expand on the theoretical proposition that mindful organizing, which is based on high-reliability organizations’ principles, can be the better fit for healthcare settings [12]. The notion of the proposed manifestation of mindful organizing practices in healthcare occupational settings is justified through our findings, as high-reliability organization principles are important to establish a safe healthcare environment [12].

Regarding the impact of sub-dimensions of workforce agility, i.e., proactivity, adaptability, and resilience, they all had a significant and positive impact on mindful organizing. These findings are consistent with the theoretical assumptions, i.e., if individuals are proactive about signs, clues, and their surroundings, they are more likely to engage in mindful organizing [9,25]. The individual’s way of doing things should be adaptable, based on contemporary conditions instead of orthodox structures. Additionally, organizations can be more reliable if they can be adaptable through their workforce collectively [38,39]. Further, the resilience of the workforce is pivotal and needs to be maintained and developed constantly while facing stressful scenarios for mindful organizing [11]. Based on the findings, there is statistical rationale to state that to initiate mindful organizing among employees, it is important to make them more proactive, adaptable, and resilient. Proactiveness amongst employees can be enhanced by encouraging employees to give more suggestions and initiate new ideas by increasing their role breadth through self-efficacy and providing more role flexibility [40,41]. Further, the literature on adaptability also states that adaptability can be further enhanced by introducing employees to unknown situations, new environments, people, and even new types of equipment in the form of training and controlled environments [42,43]. Adaptable performance is defined as one’s effective learning and behavioral outcome against ever-changing tasks [42], thus adaptability may help yield more mindful organizing. Adaptability becomes more crucial for the healthcare sector as this sector has to deal with extreme variability in its patients, changing technology, and rapidly expanding knowledge, etc. [26]. One of the last dimensions of the agility construct is resilience, which means the ability of the workforce to recoup and bounce back from adversities [44]. Since employee resilience is the outcome of the interplay between an individual and his/her given environment [45], their resilience can be enhanced through training and learning interventions, i.e., mimicking stress conditions (through formal drills/exercises), changing the environment (giving hypothetical situations to act), and providing them with problem-solving scenarios. Some of the recent literature also suggests that strategies such as building self-efficacy, gaining knowledge and skills, gaining psychological competency, developing coping strategies, and positive thinking can also enhance individual resilience [46,47]. Resilience is also important for healthcare sector employees as they have to face situations daily that are not normally observed by individuals in other professions [26].

The study findings further supported that mindful organizing has a direct effect on both safety compliance and safety participation, and the mediating role of mindful organizing between agility and safety participation has also been supported. The direct effect of mindful organizing is consistent with the prior literature [13,16,17], which provides further efficacy to the predictability of mindful organizing for safety performance. Here, it is worthwhile to mention that the number of hazards that prevail in different forms for healthcare workers at their workplace is enormous, e.g., chemical, biological, radiological, and physical. Therefore, the safety of the healthcare workforce at their workplace is not only pivotal for themselves, but also for the patients they are looking after [48]. This implies that caring for one’s self may lead to caring for another and it may also help to provide active monitoring and quality care to the patients. Our findings complement the assumptions that are necessary to develop mindful organizing amongst individuals and teams. In the light of past literature, it is essential for organizations to achieve mindful organizing and to become more reliable in their operations not only for their employees, but to avoid mishaps towards their patients as well as safeguard the health and safety of their workforce. It would be helpful to establish mindful organizing amongst teams, which then will foster positive safety behavior for nursing staff.

Previously, no studies have placed workforce agility as an antecedent for mindful organizing, thus our findings are of great theoretical contribution. Additionally, the quantitative evidence for testing objective indicators of safety performance in a healthcare setting with a multilevel construct is deficient, thus making this study unique and intriguing. Organizations can increase or generate mindful organizing among their teams through fostering the proactivity, adaptability, and resilience of their workforce. Moreover, through the utilization of mindful organizing, managers of healthcare organizations can also help identify training needs for their staff to enhance their cooperation and see which aspect is lacking to improve the safety of the staff as well as their patients.

### Study Limitation and Future Research

There is no doubt that every study has certain limitations attached to it. One of the first limitations for this study is the utilization of cross-sectional data, which itself is time-dependent, whereas a longitudinal study involving multiple periods would possibly yield some different findings. Some of the past studies have used multiple periods to test mindful organizing in high-reliability organizations (HROs), hence necessitating the need to undertake a longitudinal study in the future in the healthcare context. Secondly, in our study, the data collected from respondents through a self-reported instrument may have an impact on how honest the respondents were when answering the questionnaire [49]. This is especially crucial given that all of the staff were from private hospitals, where overall safety is emphasized, so participants may have succumbed to social desirability bias and overestimated their levels of mindful organizing. The aforementioned social desirability bias has also been discussed in the prior literature in connection with mindful organizing [13].

Further, our findings are relevant to the Malaysian cultural context, which requires future validation globally. Further, our respondents are nursing staff, who are directly providing healthcare facilities in different teams, whereas in the future, the inclusion of higher hierarchy and top management from the healthcare sector would produce interesting insights. Moreover, as we have found mediation evidence between Adaptability → Mindful Organizing → Safety Participation and Resilience → Mindful Organizing → Safety Participation, further research in different contexts comprising diversified individuals may also be expected to yield different outcomes.

## Figures and Tables

**Table 1:** Convergent validity test.

Construct	Proactivity													CR	AVE
Items	PR11	PR10	PR9	PR8	PR7	PR6	PR5	PR4	PR3	PR2	PR1			0.97	0.72
SFL→	0.84	0.84	0.83	0.85	0.84	0.86	0.83	0.87	0.86	0.85	0.85				
Mean→	3.38	3.37	3.41	3.48	3.40	3.40	3.42	3.32	3.49	3.41	3.38				
Std. Dev	1.74	1.72	1.73	1.70	1.72	1.73	1.73	1.72	1.69	1.72	1.73				
Construct	Adaptability													0.95	0.61
Items	AD13	AD12	AD11	AD10	AD9	AD8	AD7	AD6	AD5	AD4	AD3	AD2	AD1		
SFL→	0.78	0.77	0.79	0.79	0.80	0.75	0.78	0.77	0.77	0.79	0.77	0.78	0.79		
Mean→	3.22	3.30	3.38	3.36	3.33	3.36	3.33	3.25	3.29	3.34	3.28	3.31	3.27		
Std. Dev	1.47	1.51	1.50	1.49	1.51	1.55	1.49	1.49	1.50	1.50	1.49	1.50	1.47		
Construct	Resilience													0.94	0.57
Items	RS12	RS11	RS10	RS9	RS8	RS7	RS6	RS5	RS4	RS3	RS2	RS1			
SFL→	0.78	0.75	0.73	0.76	0.79	0.74	0.74	0.73	0.74	0.74	0.75	0.76			
Mean→	2.84	2.88	2.98	2.92	2.96	2.98	2.97	2.86	2.99	2.87	2.91	3.01			
Std. Dev	1.64	1.69	1.71	1.67	1.62	1.72	1.70	1.70	1.72	1.69	1.69	1.71			
Construct	Mindful Organizing													0.93	0.58
Items	MO9	MO8	MO7	MO6	MO5	MO4	MO3	MO2	MO1						
SFL→	0.84	0.84	0.83	0.85	0.84	0.86	0.83	0.87	0.86						
Mean→	3.70	3.79	3.84	3.76	3.79	3.78	3.71	3.77	3.80						
Std. Dev	1.35	1.35	1.32	1.34	1.34	1.33	1.35	1.36	1.36						
Construct	Safety Compliance													0.84	0.63
Items	SC3	SC2	SC1												
SFL→	0.82	0.82	0.74												
Mean→	3.00	2.99	2.92												
Std. Dev	1.64	1.62	1.60												
Construct	Safety Participation													0.88	0.70
Items	SC3	SC2	SC1												
SFL→	0.86	0.83	0.83												
Mean→	2.62	2.60	2.54												
Std. Dev	1.43	1.44	1.37												

Note: SFL = standardized factor loadings; CR = construct reliability; AVE = average variance extracted.

**Table 2:** Discriminant validity results.

Constructs	CR	AVE	MSV	MaxR (H)	Proactivity	Adaptability	Resilience	Mindful Organizing	Safety Compliance	Safety Participation
Proactivity	0.97	0.72	0.46	0.966	0.847					
Adaptability	0.95	0.61	0.07	0.953	−0.004	0.778				
Resilience	0.94	0.57	0.03	0.94	−0.158 **	0.013	0.752			
Mindful Organizing	0.93	0.58	0.14	0.926	0.304 ***	0.105 †	0.138 *	0.762		
Safety Compliance	0.84	0.63	0.31	0.844	0.412 ***	0.259 ***	0.085	0.368 ***	0.796	
Safety Participation	0.88	0.7	0.46	0.878	0.678 ***	0.081	−0.04	0.341 ***	0.554 ***	0.839

CR = construct reliability; AVE = average variance extracted; MSV = Maximum Shared Variance; MaxR (H) = McDonald Construct Reliability, * = *p* < 0.05. ** = *p* < 0.01, *** = *p* < 0.001, † = *p* < 0.100.

**Table 3:** Fitness indices for the measurement models.

	Absolute Fitness Indices		Incremental Fitness Indices				Parsimonious Fitness Indices
Models	RMSEA	GFI	AGFI	NFI	TLI	CFI	χ^2^/DOF
Model of Workforce Agility	0.018	0.912	0.901	0.938	0.993	0.993	1.113
Model of Mindful Organizing	0.032	0.978	0.963	0.98	0.99	0.995	1.367
Model of Safety Performance	0.001	0.997	0.991	0.997	1.01	1.000	0.472

Note: RMSEA = root-mean-square error of approximation; GFI = goodness-of-fit index; AGFI = adjusted goodness-of-fit index; NFI = normed fit index; TLI = Tucker–Lewis index; CFI = comparative fit index; PFI = parsimonious normed-fit index; χ^2^ (i.e., χ^2^/degree of freedom).

**Table 4:** Path coefficient of the final model.

Hypotheses	Estimate	S.E.	C.R.	*p*	Result
(H1) Proactivity → Mindful Organizing	0.24	0.04	6.039	***	Supported
(H2) Adaptability → Mindful Organizing	0.092	0.047	1.983	0.047	Supported
(H3) Resilience → Mindful Organizing	0.156	0.045	3.483	***	Supported
(H4) Proactivity → Safety Compliance	0.303	0.048	6.367	***	Supported
(H7) Proactivity → Safety Participation	0.549	0.046	11.895	***	Supported
(H5) Adaptability → Safety Compliance	0.239	0.053	4.486	***	Supported
(H8) Adaptability → Safety Participation	0.075	0.046	1.63	0.103	Not Supported
(H6) Resilience → Safety Compliance	0.104	0.05	2.1	0.036	Supported
(H9) Resilience → Safety Participation	0.044	0.044	0.995	0.32	Not Supported
(H10) Mindful Organizing → Safety Compliance	0.249	0.065	3.826	***	Supported
(H11) Mindful Organizing → Safety Participation	0.158	0.057	2.77	0.006	Supported

Note: *** = *p* < 0.001, Estimate = standardized regression coefficients, S.E. = standardized error, C.R. = critical ratio.

**Table 5:** Standard direct and indirect effects for the mediation model.

Mediation Effect	Direct Effect X → Y	Indirect Effect	Result
Proactive → Mindful Organizing → Safety Compliance	0.372 **	0.073 **	Partial Mediation
Proactive → Mindful Organizing → Safety Participation	0.648 **	0.045 *	Partial Mediation
Adaptability → Mindful Organizing → Safety Compliance	0.237 **	0.023 *	Partial Mediation
Adaptability → Mindful Organizing → Safety Participation	0.177 (ns)	0.014 *	Full Mediation
Resilience → Mindful Organizing → Safety Compliance	0.112 *	0.041 *	Partial Mediation
Resilience → Mindful Organizing → Safety Participation	0.045 (ns)	0.025 *	Full Mediation

(ns) = *p* > 0.05 (not significant), * = *p* < 0.05 (significant), ** = *p* < 0.01 (significant), X = (dependent variable), Y = (dependent variable).

## Data Availability

Data can be shared through the corresponding author upon reasonable request and consideration.
